# Breast Cancer Screening Recommendations for Transgender and Gender Diverse Patients: A Knowledge and Familiarity Assessment of Primary Care Practitioners

**DOI:** 10.1007/s10900-023-01228-2

**Published:** 2023-05-23

**Authors:** Evelyn F. Carroll, Genevieve A. Woodard, Colt M. St Amand, Caroline Davidge-Pitts

**Affiliations:** 1 Department of Radiology, Mayo Clinic, 200 First Street SW, Rochester, MN 55905 USA; 2 Department of Radiology, New York University Langone Health, New York, NY USA; 3 Department of Radiology, University of North Carolina, Chapel Hill, NC USA; 4 Department of Family Medicine, Mayo Clinic, Rochester, MN USA; 5 Division of Endocrinology, Diabetes and Metabolism, Mayo Clinic, Rochester, MN USA; 6 Department of Psychology, University of Houston, Houston, TX USA

**Keywords:** transgender, breast cancer, breast cancer screening, primary care

## Abstract

Breast cancer screening recommendations for transgender and gender diverse (TGD) patients have only been recently developed and many primary care practitioners (PCPs) are unaware of these specific recommendations. The aim of this study is to assess the level of familiarity and knowledge PCPs have with breast cancer screening recommendations for TGD patients. An anonymous survey was distributed to primary care physicians, primary care advanced practice practitioners, and internal medicine and family medicine residents at three academic medical systems in the United States (Mayo Clinic, University of Michigan, University of Texas – Medical Branch). Survey questions assessed the familiarity and knowledge base of TGD breast cancer screening recommendations, training and experience with TGD patients, and basic demographics of the practitioners. Of the 95 survey respondents, only 35% of respondents were aware that breast cancer screening recommendations for TGD patients existed. PCPs who had increased transgender specific health care training and direct clinical exposure to TGD patients demonstrated significantly higher levels of screening recommendation awareness. Two-thirds of respondents received TGD specific medical education during training or medical career and those who had increased transgender specific medical education or direct clinical exposure to TGD patients demonstrated significantly higher levels of screening recommendation awareness. Awareness of breast cancer screening recommendations for TGD patients is low among PCPs and varied based on the practitioner’s prior TGD education and experience. Up-to-date breast cancer screening recommendations for TGD patients should be readily available across multiple platforms, target key audiences, and integrated into transgender health educational curriculums to maximize awareness of these important recommendations.

## Introduction

Approximately 0.5% of adults in the United States identify as transgender [1]. Transgender and gender diverse (TGD) people have a gender identity which differs from their sex designated at birth. Cisgender people have a gender identity which aligns wither their designated birth sex. A subset of TGD individual will undergo medical and/or surgical gender transition, which can include gender affirming hormone therapy (GAHT) and gender embodiment surgeries such as genital reconstruction surgery (vaginoplasty, phalloplasty, or orchiectomy), chest/breast surgery (chest masculinization surgery or breast augmentation), facial surgery, and body contouring surgery. In the 2015 United States Transgender Survey, 36% of transgender men had undergone chest surgery reduction or reconstruction and 61% desired this procedure [2]. In transgender women, 11% had undergone breast augmentation and 40% desired this procedure.

TGD patients face many barriers to accessing proper health care such as refusal of service, verbal harassment, and lack of practitioner knowledge and experience [2]. A solution to alleviate one of the many barriers to care for TGD patients in the health care system is to improve health care practitioner knowledge and experience. TGD patients who perceive their health care practitioners are knowledgeable about transgender health are more likely to seek medical attention [3]. However, medical school and residency curriculum is lacking in content of care of the TGD person [4, 5]. One suspected knowledge gap for health care practitioners taking care of TGD patients is the breast cancer screening recommendations for this population.

The influence of GAHT and gender embodiment surgery on the development of breast cancer is poorly understood. In transgender women, estrogen therapy results in the development of breast tissue which is histologically identical to cisgender females [6]. Both benign and malignant pathologic processes can develop in this breast tissue [7–9]. The incidence of breast cancer in transgender women on GAHT is unknown, but likely lies between that of cisgender women and cisgender men, as demonstrated in a Dutch cohort of 3489 transgender patients, which found a 47-fold higher risk of invasive breast cancers compared to cisgender men but a 0.3-fold lower risk compared to cisgender women [10]. In transgender men, the effects of testosterone are less clear but do not appear to increase the risk of breast cancer and may lead to a decrease in breast cancer incidence [11, 12]. In transgender men who have undergone chest masculinization surgery, the risk of breast cancer in the mastectomy bed is not well established, particularly as there are varying amounts of residual breast parenchyma following chest masculinization surgery [13].

Breast cancer screening recommendations for TGD patients are based on the patient’s sex designated at birth, risk factors, use of exogenous hormones, and status of gender affirming breast/chest surgery. The incidence of breast cancer in the transgender population is largely unknown and clinical practice recommendations for TGD breast cancer screening are based primarily on several retrospective cohort studies, case reports, and consensus expert opinion. Multiple recommendations for breast cancer screening exist from the Endocrine Society, Fenway Health, University of California San Francisco (UCSF) Center of Excellence for Transgender Health, the American College of Radiology (ACR), World Professional Association of Transgender Health (WPATH), and the American College of Obstetricians and Gynecologists (ACOG) [14–19]. There is some variability in the current screening recommendations, particularly in transgender women on GAHT, amongst the noted organizations due to the the lack of robust evidence-based studies to date (Tables 1 and 2).

**Table 1:** Selected Breast Cancer Screening Recommendations for Transgender Women

Patient	American College of Radiology [17]	WPATH Standards of Care 8 [18]	USCF Center of Excellence for TGD Health [16]
Transgender women **with** 5 or more years of gender affirming hormone therapy	Annual screening mammography starting at 40 years	Same local screening recommendation as for cisgender women taking into account length of time on gender affirming hormone therapy, dosing, and age.	Biennial screening mammography starting at 50 years
Transgender women **with less** than 5 years of gender affirming hormone therapy	No screening recommended		No screening recommended
Transgender women at higher-than-average risk of breast cancer	Annual screening mammography starting at 25–30 years	Shared decision making about screening options	Not addressed

WPATH: World Professional Association of Transgender Health

UCSF: University of California San Francisco

TGD: transgender and gender diverse

MRI: magnetic resonance imaging

**Table 2:** Selected Breast Cancer Screening Recommendations for Transgender Men

Patient	American College of Radiology [17]	WPATH Standards of Care 8 [18]	USCF Center of Excellence for TGD Health [16]
Transgender men **with** chest masculinization surgery	-No screening recommended -Consider yearly chest exams	Shared decision making about screening options	Engage in dialogue about unknown risks
Transgender men **without** chest masculinization surgery	Same screening recommendation as for cisgender women	Same local screening recommendation as for cisgender women based on risk profile	Same screening recommendation as for cisgender women
Transgender men at higher-than-average risk of breast cancer and **without** chest masculinization surgery	-Annual screening mammography starting at 25–30 years - Consider screening breast MRI		Not addressed

**Table 3:** Respondent Demographics and Training

	Frequency (n)	Percent (%)
Age (years), *n = 75*		
21–30 31–40 41–50 51–60 > 60	16 21 25 7 6	21 28 33 9 8
Current Experience Level, *n = 75*		
PGY1 PGY2 PGY3 1–10 years in practice 11–20 years in practice 21 + years in practice	7 9 5 21 23 10	9 12 7 28 31 13
Medical Training Background, *n = 77*		
Family Medicine Internal Medicine or Medicine/Pediatrics Advanced Practice Practitioner	41 27 9	53 35 12
LGBTQI + Community Member, *n = 74*		
Yes No	11 63	15 85
Sex Designated at Birth, *n = 75*		
Female Male	54 21	72 28
Gender Identity, *n = 76*		
Woman Man Non-Binary	56 20 0	74 26 0
Previously Cared for Transgender or Gender Diverse Patients (n), *n = 77*		
None 1–5 6–10 11–20 >20	4 41 20 6 6	5 53 26 8 8
Transgender Specific Education (hours), *n = 76*		
None 1–5 6–10 10+	14 40 10 12	18 53 13 16
Prior Educational Types (choose all that apply), *n = 95*		
Didactic lectures Online resources Online physician message boards/social media groups Direct clinical experience as a trainee CME course in transgender health Academic transgender health literature Not applicable or none indicated	47 13 2 17 21 14 32	49 14 2 18 22 14 34

n: total number of responses per question

PGY: post graduate year

LGBTQI+: lesbian, gay, bisexual, transgender, queer, intersex, and more

CME: continuing medical education

**Table 4:** Breast Cancer Screening Recommendations Familiarity

	Frequency (n)	Percent (%)
Aware of Transgender Patient Recommendations, *n = 93*		
Yes No	33 60	35 65
Recognized Transgender Patient Resources (choose all that apply), *n = 95*		
University of California San Francisco Endocrine Society American College of Radiology Fenway Health American College of Obstetrics and Gynecology Other Not aware of any of these resources or none selected	15 8 6 3 23 7 54	16 8 6 3 24 7 57

n: total number of responses per question

**Table 5:** Screening Recommendations Confidence Level

	Frequency (n)	Percent (%)
*Rate how confident you are with the recommended breast cancer screening* recommendations *for each patient example*:		
Transgender women on gender affirming hormone therapy, *n = 85*		
Not confident at all Somewhat confident Completely confident	58 21 6	68 25 7
Transgender women NOT on gender affirming hormone therapy, *n = 82*		
Not confident at all Somewhat confident Completely confident	36 36 10	44 44 12
Transgender men who have undergone chest masculinization surgery only, *n = 83*		
Not confident at all Somewhat confident Completely confident	42 31 10	51 37 12
Transgender men on gender affirming hormone therapy but WITHOUT chest masculinization surgery, *n = 84*		
Not confident at all Somewhat confident Completely confident	48 26 10	57 31 12
Transgender men WITHOUT chest masculinization surgery and NOT on gender affirming hormone therapy, *n = 83*		
Not confident at all Somewhat confident Completely confident	30 26 27	36 31 33

n: total number of responses per question

**Table 6:** Screening Recommendations Knowledge Test Responses

	Frequency (n)*	Percent (%)
*Respondents asked to indicate if breast cancer screening mammography would be clinically appropriate for each of the following patient cases, assuming average-risk patients. † Indicates correct answer.*		
57-year-old **transgender woman** who has been on gender affirming hormone therapy for 4 years? *n = 79*		
Yes No†	51 28†	65 35
*35% correct response rate*		
38-year-old **transgender woman** who has been on gender affirming hormone therapy for 18 years? *n = 79*		
Yes No†	54 25†	68 32
*32% correct response rate*		
61-year-old **transgender woman** who has never initiated gender affirming hormone therapy due to significant medical comorbidities? *n = 79*		
Yes No†	8 71†	10 90
*90% correct response rate*		
53-year-old **transgender man** who previously underwent chest masculinization surgery? *n = 79*		
Yes No†	29 50†	37 63
*63% correct response rate*		
60-year-old **transgender man** who has NOT undergone chest masculinization surgery but has been on gender affirming hormone therapy for 25 years? *n = 79*		
Yes† No	76† 3	96 4
*96% correct response rate*		
54-year-old **transgender man** who has never had chest masculinization surgery? *n = 79*		
Yes† No	76† 3	96 4
*96% correct response rate*		
51-year-old **non-binary patient, male sex designated at birth**, who has been on hormone therapy for 10 years? *n = 79*		
Yes† No	62† 17	78 22
*78% correct response rate*		

n: total number of responses per question

**Table 7:** Future Transgender Training Experience

	Frequency (n)*	Percent (%)
Importance of Transgender-Specific Training for Primary Care Practitioners, *n = 78*		
Very important Somewhat important Somewhat not important Not important at all	57 18 2 1	73 23 3 1
Desired Future Training Resources (choose all that apply), *n = 95*		
Didactic Lectures Online Resources Online physician message boards/social media groups CME course in transgender health Academic transgender health literature None indicated	49 54 12 49 27 18	52 57 13 52 28 19
Barriers to Receiving Transgender-specific training and education (choose all that apply), *n = 95*		
Lack of faculty with expertise in this area Lack of educational content in medical training Poor direct clinical exposure in training Other None indicated	57 45 59 14 18	60 47 62 15 19

n: total number of responses per question

Primary care practitioners (PCPs) are typically the health care practitioners who help guide and recommend cancer related screening for patients. Knowledge and familiarity of cancer screening recommendations for the cisgender population, specifically breast cancer screening recommendations, is likely very high among PCPs as this is an integral part of preventative medicine. Quantification of PCP’s knowledge and familiarity of breast cancer screening recommendations in the transgender population has not been published to date, but is hypothesized to be low due to the variability of medical training curricula and content in the care of the TGD patient. The primary aim of this study is to assess the level of familiarity and knowledge PCPs have with breast cancer screening recommendations for TGD patients, and if there is variability of these levels based on the PCPs prior education and experience.

## Materials and Methods

### Study Design

The Mayo Clinic Institutional Review Board (IRB) exempted from review this cross-sectional survey of PCPs. An anonymous electronic survey was distributed to primary care internal medicine and family medicine physicians, and primary care advanced practice practitioners (APPs) at all Mayo Clinic enterprise sites (Rochester, MN; Jacksonville, FL; Phoenix/Scottsdale, AZ, and the Mayo Clinic Health System in Minnesota and Wisconsin). Additionally, the anonymous survey was sent to all participating internal medicine and family medicine residencies in the Mayo Clinic enterprise, the University of Michigan internal medicine residency, and University of Texas – Medical Branch family medicine residency. The sites were chosen primarily because a point person was identified at each site to help distribute the survey to practitioners, and to increase diverse representations of geographic, training, and experience level. Surveys were distributed via electronic mail utilizing the research electronic data capture tool (REDCap) hosted by Mayo Clinic. All data was collected between March and June 2022.

### Survey Design

The 25-question survey collected demographics, training, familiarity and knowledge of TGD breast cancer screening recommendations, desired future TGD training and potential barriers. To incentivize completion of the survey, respondents received the correct answers to the knowledge base question section (answers based upon consensus from all the major medical organizations that have published TGD breast cancer screening recommendations to date) and TGD health resource links, including TGD breast cancer screening recommendations, upon completion of the survey.

### Statistical Analysis

Descriptive statistics were reported as frequency (n) and percent (%). Fischer exact tests were performed to compare distributions of categorical variables. Statistical analyses were completed with SAS OnDemand for Academics. Two-sided p-values < 0.05 were considered statistically significant.

## Results

### Respondents Demographics and Training

A total of 95 PCPs responded to the TGD breast cancer survey from a pool of 700 potential respondents, for a 14% response rate. Of those who responded, 69% (66/95) fully completed the survey with 30% (29/95) leaving some questions unanswered. The age groups for respondents spanned from 21 to 30 years of age to greater than 60 years, with almost half of the practitioners 41 years of age or older (51%, 38/75; Table 3). More respondents (72%, 54/75) indicated they were attendings or APPs than trainees (28%, 21/75). Almost half of respondents were family medicine (53%, 41/77), while 35% (27/77) were internal medicine or medicine/pediatrics, and 12% (9/77) were APPs. 15% (11/74) of respondents self-identified as an LGBTQI + community member and 74% (56/76) of respondents had a female gender identity.

The amount of TGD specific education received in training and/or career ranged from none to 10 or more hours, with 29% (22/76: Table 3) reporting 6 or more hours. The majority (95%, 73/77) of practitioners reported they had cared for at least one, and 42% reported they had cared for 6 or more TGD patient during their practice or training). Additionally, approximately two thirds (66%, 63/95) of practitioners received TGD specific medical education during training and/or career with didactic lectures being most common (49%, 47/95).

### Transgender Breast Cancer Screening Recommendation Familiarity

Overall, 35% (33/93; Table 4) of survey responders were aware that breast cancer screening recommendations for transgender patients existed. Only 43% (41/95) of respondents indicated they were familiar with common resources to find recommendations pertaining to breast cancer screening in TGD patients. Of the practitioners who were aware that breast cancer screening recommendations for TGD patients existed, the UCSF recommendations were the most reported resource (36%, 12/33).

Practitioners with 6 or more hours of prior TGD specific education in training and/or career more often reported awareness of these screening recommendations 67% (14/21) than those who had received only 0–5 h of education (28% 15/53, p < 0.004). Similarly, awareness of these recommendations was significantly higher among those who had cared for more TGD patients in training and/or career (6 + TGD patients: 55%, 17/31 versus 0–5 TGD patients: 27%, 12/44, p < 0.03). Respondents who identified as LGBTQI + community members versus nonmembers, were also significantly more aware of the recommendations (72%, 8/11 versus 33%, 20/61, p < 0.02).

### Transgender Women Screening Recommendation Confidence and Knowledge Test Responses

Practitioners were asked to rate their personal confidence levels with breast cancer screening recommendations for specific TGD patient case-based scenarios (Table 5). Just under a third (32%, 27/85) were somewhat to completely confident with recommendations for average-risk transgender women on GAHT. These lower confidence levels matched the PCP’s abilities to correctly identify that breast cancer screening was NOT indicated for a 57-year-old transgender woman on GAHT for 4 years, with only 35% (28/79) responding correctly on the knowledge test portion of the survey (Table 6). And, only 32% (25/79) of respondents correctly answered that a 38-year-old transgender woman on GAHT for 18 years should NOT be screened for breast cancer.

Over half (56%, 46/82) of practitioners were somewhat to completely confident with recommendations for transgender women NOT on GAHT (Table 5), and their ability to correctly answer the case-based knowledge scenarios of transgender women patients NOT on GAHT was higher than these confidence levels. Most respondents (90%, 71/79) correctly identified that a 61-year-old transgender woman NOT on GAHT secondary to comorbidities should NOT be screened (Table 6).

### Transgender Men Screening Recommendation Confidence and Knowledge Test Responses

For average-risk transgender men who had undergone chest masculinization surgery, approximately half (49%, 41/83) of respondents were somewhat to completely confident with breast cancer screening recommendations (Table 5), but a slightly higher correct response rate of 63% (50/79) was observed for the knowledge case-scenario: A 53-year-old transgender man with prior history of chest masculinization surgery should NOT undergo cancer screening (Table 6).

Less than half (43%, 36/84; Table 5) of respondents felt somewhat to completely confident with breast cancer screening recommendations for transgender men on GAHT who had NOT undergone chest masculinization surgery. Despite these lower confidence levels, the majority (96%, 76/79) correctly identified that a 60-year-old transgender man on GAHT and WITHOUT prior chest masculinization surgery should be screened for breast cancer (Table 6).

### Future Transgender Training Experience

When asked about the importance of TGD specific training for PCPs, most respondents indicated it was very important (73%, 57/78; Table 7). This was significantly more (86%) among practitioners ages 21–40 years versus 41 + years (61%, p = 0.02).

Over half of practitioners identified didactic lectures (52%, 49/95), online resources (57%, 54/95), and CME courses (52%, 49/95) as the top desired resources to better educate on this topic in the future. While academic health literature was the second lowest desired resource for educating oneself overall, it was statistically significantly more desired amongst trainees, with 67% (14/21) of PGY1-3 versus 24% (13/54) of attendings and APP (p = 0.001).

While there are many barriers to receiving TGD specific training and education, lack of faculty with expertise in this area (60%, 57/95), and poor direct clinical exposure in training (62%, 59/95) were the most frequently indicated reasons. When assessed by age, 89% (33/37) of those ages 21–40 years versus 68% (26/38) who are 41 years or older indicated poor direct clinical exposure in training as a barrier in receiving transgender-specific training and education (p < 0.05).

## Discussion

This study revealed a lack of PCP familiarity and knowledge of breast cancer screening in TGD patients, and also demonstrated insightful patterns on prior transgender health training and educational experiences within this cohort of respondents. Lack of practitioner knowledge with regards to TGD health is a known barrier to achieving optimal and equitable health care for TGD patients [2]. PCPs play an important role in ensuring all patients adhere to recommended cancer screening recommendations to prevent potential morbidity and mortality.

In order to achieve adequate knowledge and familiarity with a specific health care topic, a clinician must be at least be aware of potential resources to refer to when the appropriate clinical scenario arises. In the case of breast cancer screening in TGD patients, only 35% were aware that recommendations even existed, let alone where to find them. These findings are in keeping with previous study results in other specialties. For example, 41% of obstetrics and gynecology practitioners knew the recommendations of breast cancer screening in transgender women [20]. Similarly, a survey of breast imaging radiologists in 2022 found that only 38% of respondents follow breast cancer screening recommendations or provide screening recommendations for transgender women on GAHT in their practice [21]. The percentage of PCPs who were aware of breast cancer screening recommendations exist for the TGD population increased with 6 or more hours of transgender specific education and previously caring for 6 or more TGD patients, suggesting that increased transgender specific education and clinical experience is important for developing better awareness of these recommendations.

In the knowledge assessment portion of this survey pertaining to transgender women, respondents struggled with case examples of transgender women on GAHT, likely because of age specific and GAHT duration specific recommendations. Specifically, less than one third of respondents correctly identified that a young transgender woman who had been on GAHT for nearly her entire adult life, but was still below the age of 40, did not need to start screening mammogram until she reached at least 40 years of age. Likewise, only 35% of respondents correctly identified that despite the older age of a 57-year-old transgender woman, her duration of GAHT was less than 5 years, and therefore she did not yet need to begin screening mammography for another year.

Respondents fared comparatively more strongly with regards to the knowledge assessment of breast cancer screening recommendations in transgender men without chest masculinization surgery, with over 90% correctly responding to these patient’s meeting screening criteria. However, over a third (37%) incorrectly chose to perform screening mammography in an example of a 53-year-old transgender man who had previously undergone chest masculinization surgery. The chest masculinization technique is different from that of an oncologic bilateral mastectomy, and the cancer risk of residual breast tissue left to contour the chest is unclear [13, 22]. There are no studies currently to support image-based screening for these patients beyond physical exam, which is based upon guidelines from high-risk cisgender women with prior mastectomies [21, 22].

Several important trends about TGD health experience and education emerged from assessing various subgroups of respondents in this survey. Younger practitioners (21–40 years) place a higher importance on TGD specific educational training, possibly because they report higher rates of direct clinical experience with TGD patients as a trainee. However, a lack of faculty with expertise and poor direct clinical exposure in training were reported as the most frequent barriers to receiving TGD specific training and education, which was significantly higher for younger respondents (21–40 years). These results suggest medical trainees should have the opportunity to rotate through transgender or LGBTQI + specific clinics, which are often staffed with faculty who have expertise with TGD patients and offer higher volumes of TGD patients. Respondents who identified as a member of the LGBTQI + community demonstrated increased awareness of TGD breast cancer screening recommendations compared to their respective non-LGBTQI + counterparts, possibly due to an increased interest in TGD health and caring for more TGD patients.

### Limitations

This study has several limitations, including its cross-sectional survey design and small sample size secondary to a low response rate (14%). However, those who did response were a diverse sample of trainees and practitioners, from family medicine to internal medicine and including APP, and across a wide range of age-groups and years of experience. Furthermore, among the respondents there was diverse representation of gender identity and both LGBTQI + and non-LGBTQI + community members. Although demographic data was collected for survey respondents, it was not collected for non-respondents. The survey invitation email was titled “Transgender Breast Cancer Screening Survey.” Practitioners and trainees who chose not to complete the survey may in part have felt the survey topic did not pertain to them because they do not routinely care for TGD patients or they were not comfortable with TGD patient care. Alternatively, practitioners and trainees with an interest in TGD patient care may have been more enthusiastic about completing the survey. Our survey findings likely overestimate the average level of knowledge and familiarity of breast cancer screening recommendations in TGD patients for PCPs, supporting the need to improve TGD specific medical education further. Survey questions did not require an answer before moving onto the next question in order to decrease the burden on the respondent, resulting in missing data for some questions and the potential for unknown bias. Despite these limitations, patterns in prior TGD education and desired future resources for breast screening recommendations for TGD patients were observed, which may aid in development of TGD curriculum for trainees and continuing education tools for practitioners.

## Conclusion

Awareness of breast cancer screening recommendations for TGD patients is low among PCPs, and the majority of respondents did not feel confident about their knowledge of screening recommendations. PCPs who had increased transgender specific health care training and direct clinical exposure to TGD patients, demonstrated significantly higher levels of awareness of these recommendations. Breast cancer screening recommendations for TGD patients will continue to evolve as ongoing research shapes the evidence for screening in this patient population. Up-to-date, clear, and concise recommendations should be readily available across multiple platforms, target key audiences, and integrated into transgender health educational curriculums to maximize awareness of these important recommendations and ultimately improve the care of or TGD patients.

## Abbreviations

TGD: transgender and gender diverse
GAHT: gender affirming hormone therapy
LGBTQI: lesbian, gay, bisexual, transgender, queer, intersex
UCSF: University of California San Francisco
ACOG: American College of Obstetricians and Gynecologists
WPATH: World Professional Association of Transgender Health
ACR: American College of Radiology
APP: advanced practice practitioner
PGY: post graduate year
PCP: primary care practitioner
